# Respiratory Syncytial Virus Hospital-Based Burden of Disease in Children Younger Than 5 Years, 2015-2022

**DOI:** 10.1001/jamanetworkopen.2024.7125

**Published:** 2024-04-18

**Authors:** Robert J. Suss, Eric A. F. Simões

**Affiliations:** 1 Department of Pediatric Infectious Diseases, University of Colorado School of Medicine and Children’s Hospital Colorado, Aurora; 2 Center for Global Health, Department of Epidemiology, Colorado School of Public Health, Aurora

## Abstract

**Importance:**

Respiratory syncytial virus (RSV) resurgences have been noted following the COVID-19 pandemic in many countries. Recent findings suggest that the 2021 and 2022 RSV seasons were more severe than in past seasons, and age distribution may have shifted toward older children in the younger than 5 years age group.

**Objectives:**

To estimate age-specific changes in RSV hospital-based burden of disease before and after the COVID-19 pandemic and to compare incidence by Medicaid use.

**Design, Setting, and Participants:**

This retrospective cohort study included children younger than 5 years diagnosed with RSV and bronchiolitis at 50 US children’s hospitals in 10 US geographic regions. The included participants had an encounter in intensive care, inpatient, emergency, or observational units, between June 1, 2015, and March 31, 2023.

**Exposures:**

Diagnosis of RSV, bronchiolitis, or both at encounter.

**Main Outcome and Measures:**

Incidence rate ratio of hospital use within each care unit before vs after the COVID-19 pandemic. It was hypothesized a priori that incidence of hospital use would increase overall in 2021 and 2022 compared with 2015 to 2019 and that the increase would be greater among children 12 months and older.

**Results:**

Of 924 061 study participants (median [IQR] age, 8 (5-16) months; 535 619 [58.0%] male), 348 077 (37.7%) were diagnosed with RSV. Of these, 187 850 (54.0%) were hospitalized. Incidence rate ratios of hospitalization increased for all ages in 2021 and 2022 compared with 2015 to 2019. Children aged 24 to 59 months were 4.86 (95% CI, 4.75-4.98) times as likely to be hospitalized in 2022 compared with 2015 to 2019, whereas infants aged 0 to 5 months were 1.77 (95% CI, 1.74-1.80) times as likely. Medicaid patients were more likely to be hospitalized than non-Medicaid patients regardless of year.

**Conclusions and Relevance:**

Hospitalizations for RSV and bronchiolitis demonstrated atypical seasonality in 2021 and 2022, with an overall increase in RSV encounters. Postpandemic RSV hospitalization increased for all ages, but especially among older children, whereas bronchiolitis hospitalization was decreased or unchanged compared with earlier seasons. These findings suggest some of the observed increase in RSV hospital use may be due to increased testing.

## Key Points

**Question:**

What is the association of the COVID-19 pandemic with respiratory syncytial virus (RSV) hospital-based burden of disease in children younger than 5 years?

**Findings:**

In this cohort study of 924 061 infants and children, RSV-related hospitalization, intensive care unit use, and short stay use were significantly higher in all age groups but highest for the 2- to 5-year age group in 2021 and 2022 compared with 2015 to 2019, whereas these encounters were equally or less likely among those diagnosed with bronchiolitis. The emergency departments saw lower rates in all but the oldest children (ie, aged 24-59 months).

**Meaning:**

These findings suggest that epidemiologic characteristics and seasonal timing of the RSV season have shifted since the COVID-19 pandemic, with older age groups being the hardest hit; however, some of these changes may be attributable to increases in testing.

## Teaser

This cohort study examines respiratory syncytial virus (RSV) among infants and children and assesses differences in hospital use overall, hospital use by care unit, and the relative burden of RSV in the Medicaid population before vs after the COVID-19 pandemic.

## Introduction

Studies outside the US in the post–COVID-19 pandemic period (ie, 2020 to the present) have demonstrated a shift in epidemiologic characteristics of the respiratory syncytial virus (RSV) season. For example, delayed RSV seasons have been observed in France,^1,2^ Germany,^3^ Finland,^4^ Israel,^5^ and England,^6^ as well as in the Southern Hemisphere, including in Argentina,^7^ South Africa,^8^ and Australia.^9,10^ In 1 study, a delayed onset of the first postpandemic RSV season was reported in 11 countries, including South Korea, Canada, Chile, Brazil, and the US, among others.^11^ Nonpharmaceutical interventions (NPIs) are generally attributed internationally for the delayed RSV resurgences.^1,4,9,10,12,13,14,15,16,17^

Some differences in epidemiologic characteristics have been noted in such resurgences. In New York, increased RSV severity among younger ages was observed,^18^ whereas a smaller resurgence was observed in France, with severity and age distribution more typical of prepandemic RSV seasons.^1^ Compared with the 2018 to 2019 and 2019 to 2020 RSV seasons in France, the 2020 to 2021 outbreaks involved more children aged 6 to 11 months but fewer younger than 6 months, as well as an increase in hospital admissions; however, a decrease in overall severity was observed.^2^ In contrast, an epidemiologic shift was reported in the US to a spring and summer peak RSV season in 2021, with no change in age distribution among patients with RSV.^14^

Taken as a whole, the literature suggests a potential shift in RSV burden toward older children since 2020. It has been suggested that pandemic disruption created an increased susceptibility among older children who have had no prior exposure to endemic viruses, including RSV.^19^ The Centers for Disease Control and Prevention (CDC) reported in early 2023 that the 2022 RSV season occurred later than that of 2021 but still earlier than in prepandemic seasons; although this may suggest a return to prepandemic seasonality, the CDC warns of potential off-season resurgences in the future.^20^

An additional consideration regarding changes in RSV seasonality is potential demographic shifts. For example, RSV incidence and severity are higher in boys than girls among pediatric populations, a trend that has been demonstrated globally.^21,22,23,24,25,26^ Additionally, Medicaid recipients have an almost twice higher rate of RSV hospitalization and higher mortality rates than privately insured children.^27,28,29,30^ However, whether the postpandemic RSV seasons affected Medicaid recipients differentially is unstudied. This study aimed to (1) estimate the relative change in incidence of hospital use for RSV from the 2015 to 2019 seasons to the 2 resurgent seasons after the COVID-19 pandemic (ie, 2021 and 2022); (2) examine the incidence of RSV hospital use by care unit (eg, intensive care unit [ICU], inpatient, emergency department [ED], and observation unit) in 4 age groups (0-5, 6-11, 12-23, and 24-59 months of age); and (3) quantify the role of the postpandemic resurgence on the relative burden of RSV in the Medicaid population.

## Methods

Patient cohorts were identified using the Pediatric Hospital Information Systems (PHIS) database from the Children’s Hospital Association. This database contains patient-level data from more than 200 member hospitals throughout the US. Two cohorts were identified using the PHIS Cohort Builder, based on *International Classification of Diseases, Ninth Revision* (*ICD-9*) and* International Statistical Classification of Diseases and Related Health Problems, Tenth Revision* (*ICD-10*) diagnostic codes, patient age at admission, and hospital. The RSV cohort was identified using *ICD-9* and *ICD-10* codes 466.11, 079.6, 480.1, J21.0, J12.1, B97.4, and J20.5; the non-RSV bronchiolitis cohort used codes 466.0, 466.19, J20.8, J20.9, J21.1, J21.8, and J21.9. Patients with a bronchiolitis diagnosis at encounter were only included in the cohort if they did not also have any RSV diagnosis (as specified above). Only patients younger than 60 months were included. Patients were categorized into 4 age groups: 0 to 5, 6 to 11, 12 to 23, and 24 to 59 months at encounter or admission. This study received approval with institutional exemption from the Colorado Multiple Institutional Review Board because it is secondary research. The study follows the Strengthening the Reporting of Observational Studies in Epidemiology (STROBE) reporting guideline for observational studies.

A hospital was included in the study if it reported data to PHIS through the first quarter of 2023 (March 31, 2023), although some hospitals may not have had data available for all years or care units of interest. A total of 50 hospitals were included in both cohorts, with patient encounters occurring between June 1, 2015, and March 31, 2023. To aggregate hospital encounters regionally, states were matched to the US Department of Health and Human Services–defined regions. This is the process by which the CDC has determined RSV seasonality in the US since 2007.^31^ Regional RSV seasonality by age and care unit was determined.

Laboratory and *Current Procedural Terminology* code databases from the PHIS were also used to obtain testing data, which were used to evaluate whether the proportion of RSV testing among the non-RSV cohort had changed over time. *Current Procedural Terminology* codes for respiratory tests that include RSV were obtained from the Centers for Medicare & Medicaid Services^32^; these codes were additionally matched to respiratory tests in the PHIS laboratory database, which is based on laboratory-specific billing codes.

### Statistical Analysis

All analyses were conducted using Stata/SE software, version 17.0 (StataCorp LLC) and 64-bit R, version 4.3.0 (with RStudio “Cherry Blossom” Release 2023-05-09 for Windows; R Foundation for Statistical Computing). Weekly aggregate counts for the RSV, non-RSV bronchiolitis, and combined RSV plus non-RSV bronchiolitis cohorts were determined by epidemiologic week for seasonality comparison. Case counts for each cohort were obtained for 4 distinct periods: June 30, 2015, to December 31, 2019 (prepandemic RSV seasonality), and calendar years January 1 through December 31, 2020, 2021, and 2022 (postpandemic RSV seasonality). The early months of 2020, which would otherwise have been included in the 2019 to 2020 RSV season, were instead included in postpandemic seasonality. This approach allowed for (1) prepandemic seasonality spanning 5 RSV seasons, except for the disruptions of early 2020, and (2) a distinction of postpandemic incidence defined as beginning on January 1, 2020, such that full calendar years of data were consistently used beginning when seasonal irregularities were first observed.

To estimate incidence, we extrapolated an annual population estimate of younger than 5 years using the population-based RSV hospitalization rates per age group reported by Rha et al^33^ for the 2015 to 2016 RSV season. These rates were applied to the number of hospitalizations in the PHIS cohort by age group to obtain a population estimate per month of age. Cumulative incidences of RSV and non-RSV bronchiolitis were then calculated for each time range by age group and care unit. Incidence of hospital admissions was calculated from combined ICU and inpatient admissions. Incidence rate ratios (IRRs) were then derived comparing 2020, 2021, and 2022 incidence with that of 2015 to 2019. A normal approximation was assumed to calculate 95% CIs for incidence and IRR, given the large sample sizes; thus, 2-sided hypothesis testing was conducted with α = .05 a priori levels of significance. These groups were further stratified by sex and use of Medicaid to estimate relative risk within each period. Patient demographic characteristics (eg, sex, race, age, care unit, and length of stay) were retrieved from the PHIS database, aggregated, and compared between cohorts. Data on race were collected because there are known disparities in RSV burden by race. Significance tests (ie, χ^2^ test, 2-sample, unpaired *t* test, or Mann-Whitney test, as appropriate) were performed to compare patient characteristics.

## Results

For the defined period between 2015 and 2022, a total of 348 077 RSV cases and 575 984 non-RSV bronchiolitis cases were identified, for a total of 924 061 patients in the combined cohort. The median (IQR) age was 8 (5-16) months, with a median (IQR) length of stay of 1 (0-2) day. A total of 535 619 patients were male (58.0%) and 388 339 (42.0%) were female. A significantly higher proportion of patients with RSV were admitted to the ICU (68 550 [19.7%]) than patients with non-RSV bronchiolitis (49 560 [8.6%]). Of the 924 061 total patients, 3604 (0.4%) were American Indian or Alaska Native, 25 069 (2.7%) were Asian, 259 275 (28.1%) were Black, 5864 (0.6%) were Native Hawaiian or Pacific Islander, 460 571 (49.8%) were White, 119 977 (13.0%) were of other races (any race not already specified), and 49 701 (5.4%) had missing race data. Data on gestational age were available for 106 851 patients (30.7%) with RSV, 161 106 patients (28.0%) with non-RSV bronchiolitis, and 267 957 (29.0%) of all patients from both cohorts. Medicaid use data were available for 915 241 (99.0%) of all patients and income data for 907 366 (98.2%). Most encounters (582 831 [63.7%]) were Medicaid insured. Data for all other characteristics analyzed were complete. The characteristics of patients with RSV and non-RSV bronchiolitis are summarized in Table 1. Regional seasonality of RSV cases by care unit are presented in eFigure 1 in Supplement 1.

**Table 1.** Characteristics of Patients Younger Than 5 Years With RSV and Bronchiolitis, 2015-2022^a^

Characteristic	RSV (n = 348 077)	Bronchiolitis (n = 575 984)	Total (N = 924 061)
Sex			
Male	192 122 (55.2)	343 497 (59.6)	535 619 (58.0)
Female	155 887 (44.8)	232 452 (40.4)	388 339 (42.0)
Unknown	68 (0.02)	35 (0.01)	103 (0.01)
Age group, mo			
0-5	147 447 (42.4)	183 063 (31.8)	330 510 (35.8)
6-11	64 934 (18.7)	170 078 (29.5)	235 012 (25.4)
12-23	69 297 (19.9)	161 639 (28.1)	230 936 (25.0)
24-59	66 399 (19.1)	61 204 (10.6)	127 603 (13.8)
Race			
American Indian or Alaska Native	1702 (0.5)	1902 (0.3)	3604 (0.4)
Asian	10 918 (3.1)	14 151 (2.5)	25 069 (2.7)
Black	70 307 (20.2)	188 968 (32.8)	259 275 (28.1)
Native Hawaiian or Pacific Islander	2393 (0.7)	3471 (0.6)	5864 (0.6)
White	194 043 (55.8)	266 528 (46.3)	460 571 (49.8)
Other^b^	47 608 (13.7)	72 369 (12.6)	119 977 (13.0)
Missing	21 106 (6.1)	28 595 (5.0)	49 701 (5.4)
Ethnicity			
Hispanic or Latino	86 715 (24.9)	133 497 (23.2)	220 212 (23.8)
Not Hispanic or Latino	242 142 (69.6)	416 702 (72.4)	658 844 (71.3)
Unknown	19 220 (5.5)	25 785 (4.5)	45 005 (4.9)
Unit of care			
ICU	68 550 (19.7)	49 560 (8.6)	118 110 (12.8)
Inpatient	109 457 (31.5)	96 884 (16.8)	206 341 (22.3)
ED	127 550 (36.6)	374 347 (65.0)	501 897 (54.3)
Observation unit	42 520 (12.2)	55 193 (9.6)	97 713 (10.6)
LOS, median (IQR), d	1 (0-3)	0 (0-1)	1 (0-2)
Comorbidity	48 261 (13.9)	50 923 (8.8)	99 184 (10.7)
Preterm, No./total No. reported (%)			
	29 023/106 851 (27.2)	45 202/161 106 (28.1)	74 225/267 957 (27.7)
Medicaid, No./total No. reported (%)			
	203 292/344 575 (59.0)	379 539/570 666 (66.5)	582 831/915 241 (63.7)
Median household income, mean (SD), $			
	44 597/340 508 (16 550)	42 230/566 858 (15 980)	43 118/907 366 (16 237)

Abbreviations: ED, emergency department; ICU, intensive care unit; LOS, length of stay; RSV, respiratory syncytial virus.

^a^ Data are presented as number (percentage) of patients unless otherwise indicated.

^b^ The Pediatric Hospital Information Systems database value of “other” indicates any race not reported as American Indian or Alaska Native, Asian, Black, or Native Hawaiian or Pacific Islander.

There was an overall shift in RSV seasonality toward a summer season in 2021. Although seasonal timing began to shift back to a more typical pattern (eg, occurring in colder months) in 2022 to early 2023, the number of cases in children younger than 5 years was higher overall during this season. Notably, however, the peak of the RSV season occurred much earlier in 2022 to 2023 than before 2020 (ie, from September to November rather than from December to January as previously evident). These patterns are shown in the Figure. Corresponding seasonality for the RSV and non-RSV bronchiolitis combined cohort is shown in eFigure 2 in Supplement 1. Incidence of RSV and non-RSV bronchiolitis hospital use decreased across all age groups in 2020 compared with the 2015 to 2019 seasons (Table 2). Overall, infants and children younger than 5 years were less likely to be diagnosed with bronchiolitis and more likely to be diagnosed with RSV in 2021 and 2022 compared with 2015 to 2019 across age groups (Table 3; eTables 1 and 2 in Supplement 1). In general, the IRR for the combined cohort was close to 1 in 2021 in infancy but highest in children aged 24 to 59 months. In 2022, the IRR for this combined rate significantly increased across age groups. This trend was seen across most care units (Table 3; eTable 2A-B in Supplement 1) except for outpatient departments, which primarily saw a significantly lower rate of use in infants in both 2021 and 2022 (eTable 2C-D in Supplement 1).

**Figure.**
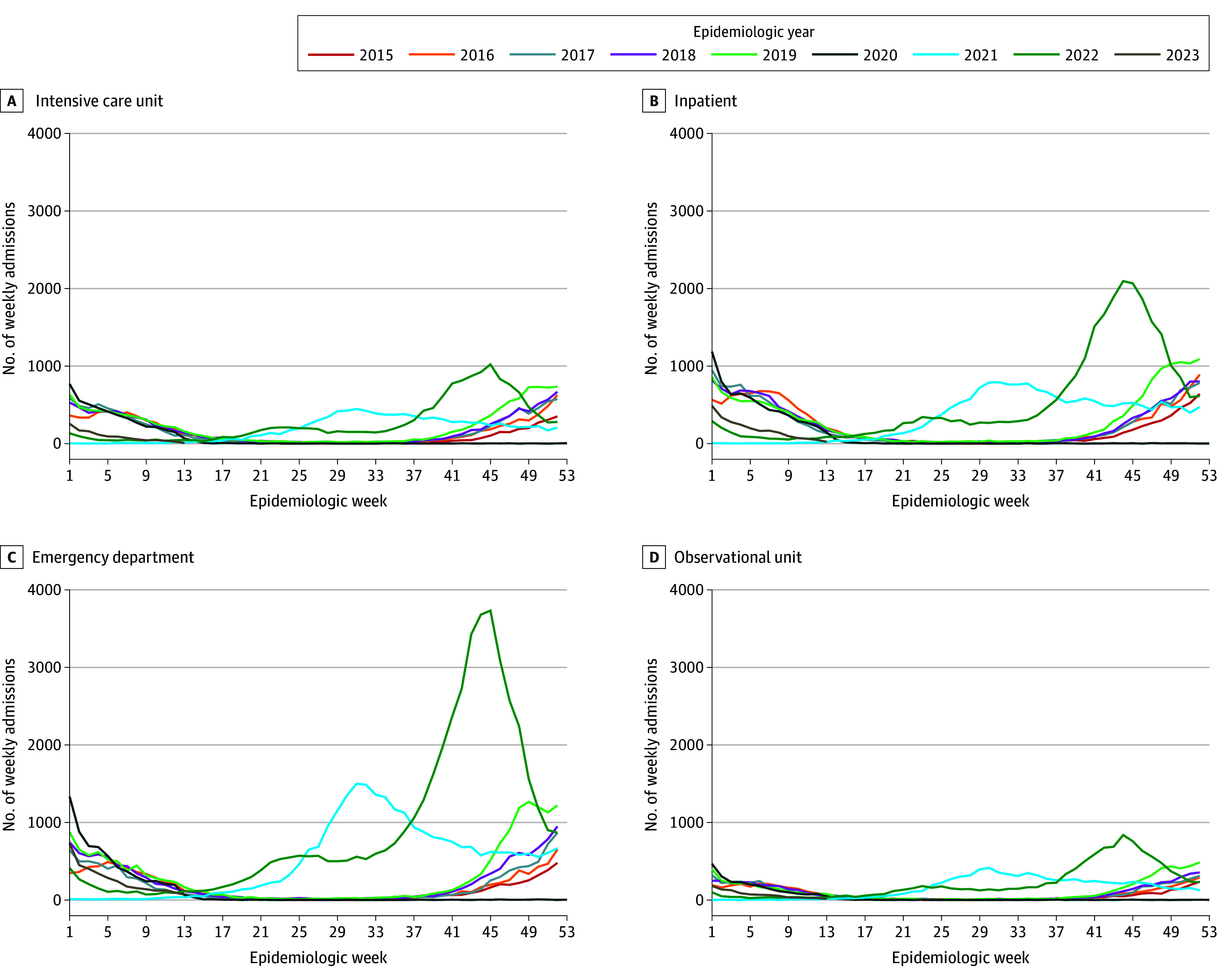
Respiratory Syncytial Virus Hospital Use in Patients Younger Than 5 Years in US Pediatric Hospital Information Systems Hospitals, 2015-2023 Data are shown through March 31, 2023. The postpandemic period includes 2020, 2021, and 2022. Epidemiologic weeks run Sunday through Saturday, with week 1 ending the first Saturday of January.

**Table 2.** Incidence of RSV and Bronchiolitis Hospitalizations in Infants and Children Younger Than 60 Months, 2015-2022^a^

Age	RSV rate per 1000 children (95% CI)				RSV and bronchiolitis rate per 1000 children (95% CI)			
	2015-2019	2020	2021	2022	2015-2019	2020	2021	2022
Age, mo								
<1	17.6 (17.5-17.6)	8.8 (8.6-9.0)	20.7 (20.6-20.9)	26.6 (26.4-26.8)	23.3 (23.2-23.4)	11.9 (11.7-12.1)	25.5 (25.4-25.7)	29.6 (29.4-29.7)
1	28.1 (28.0-28.2)	14.5 (14.3-14.7)	34.0 (33.8-34.2)	46.0 (45.9-46.2)	40.5 (40.4-40.6)	21.5 (21.3-21.7)	43.2 (43.0-43.4)	51.8 (51.6-52.0)
2	17.3 (17.3-17.4)	9.3 (9.1-9.5)	22.7 (22.5-22.9)	31.7 (31.5-31.9)	27.7 (27.7-27.8)	14.6 (14.4-14.8)	30.3 (30.1-30.4)	36.3 (36.1-36.5)
3	15.8 (15.7-15.9)	8.3 (8.0-8.5)	22.7 (22.5-22.9)	31.6 (31.4-31.8)	29.0 (28.9-29.1)	15.2 (15.0-15.4)	31.7 (31.5-32.0)	37.0 (36.8-37.2)
4	12.3 (12.2-12.4)	6.7 (6.5-6.9)	16.8 (16.5-17.0)	24.7 (24.5-24.9)	25.3 (25.3-25.4)	13.9 (13.7-14.1)	24.5 (24.3-24.7)	30.8 (30.6-31.0)
5	10.0 (9.9-10.1)	5.3 (5.1-5.5)	12.6 (12.4-12.8)	20.4 (20.2-20.6)	21.9 (21.8-22.0)	11.8 (11.6-12.0)	21.0 (20.8-21.2)	26.2 (26.0-26.4)
6	9.9 (9.8-10.0)	5.6 (5.4-5.8)	12.7 (12.4-12.9)	20.8 (20.5-21.0)	23.9 (23.8-24.0)	13.9 (13.7-14.1)	22.4 (22.2-22.6)	27.9 (27.7-28.1)
7	5.7 (5.6-5.8)	3.3 (3.1-3.5)	6.9 (6.7-7.0)	13.3 (13.1-13.5)	14.8 (14.7-14.9)	8.1 (7.9-8.2)	14.0 (13.8-14.2)	18.3 (18.1-18.5)
8	5.4 (5.3-5.5)	2.9 (2.7-3.1)	7.2 (7.0-7.4)	12.6 (12.4-12.7)	14.5 (14.4-14.6)	7.9 (7.7-8.1)	15.1 (14.9-15.2)	17.6 (17.4-17.8)
9	4.6 (4.5-4.7)	2.5 (2.3-2.7)	5.6 (5.4-5.8)	10.1 (9.9-10.3)	12.6 (12.5-12.6)	6.9 (6.7-7.0)	12.3 (12.2-12.4)	14.4 (14.3-14.5)
10	3.1 (3.1-3.2)	1.7 (1.5-1.8)	4.0 (3.8-4.1)	7.4 (7.3-7.6)	9.1 (9.1-9.2)	5.0 (4.9-5.1)	9.7 (9.5-9.8)	10.9 (10.7-11.0)
11	5.4 (5.3-5.5)	2.8 (2.6-3.0)	7.5 (7.3-7.7)	13.1 (12.9-13.3)	15.8 (15.8-15.9)	8.2 (8.1-8.4)	17.8 (17.6-17.9)	19.2 (19.0-19.4)
Age group, mo								
0-5	16.7 (15.6-17.9)	8.7 (8.7-8.8)	21.3 (21.2-21.4)	29.6 (29.6-29.7)	27.3 (27.2-27.4)	14.4 (14.2-14.6)	28.8 (28.6-29.0)	34.5 (34.3-34.7)
6-11	5.7 (5.7-5.8)	3.1 (3.1-3.2)	7.3 (7.2-7.4)	13.0 (12.9-13.1)	15.3 (15.2-15.4)	8.4 (8.2-8.6)	15.4 (15.2-15.6)	18.3 (18.0-18.5)
12-23	3.5 (3.4-3.5)	1.9 (1.8-2.0)	5.3 (5.2-5.3)	13.5 (13.4-13.6)	10.0 (9.9-10.1)	5.8 (5.6-6.0)	13.9 (13.7-14.1)	17.7 (17.5-17.9)
24-59	0.7 (0.6-0.7)	0.4 (0.3-0.4)	1.0 (1.0-1.1)	3.2 (3.2-3.2)	1.1 (1.1-1.2)	0.7 (0.6-0.7)	1.8 (1.7-1.8)	3.6 (3.5-3.7)

Abbreviation: RSV, respiratory syncytial virus.

^a^ Overall hospital admissions (intensive care unit and inpatient).

**Table 3.** Incidence Rate Ratios (95% CIs) of RSV and Bronchiolitis Hospitalizations in Infants and Children Younger Than 60 Months, 2015-2022^a^

Age	RSV			RSV and bronchiolitis		
	2020	2021	2022	2020	2021	2022
Age, mo						
<1	0.50 (0.47-0.53)	1.18 (1.13-1.23)	1.51 (1.45-1.57)	0.51 (0.48-0.54)	1.10 (1.05-1.14)	1.27 (1.22-1.32)
1	0.52 (0.49-0.55)	1.21 (1.16-1.26)	1.64 (1.58-1.69)	0.53 (0.51-0.56)	1.07 (1.03-1.10)	1.28 (1.24-1.32)
2	0.53 (0.50-0.57)	1.31 (1.25-1.37)	1.83 (1.76-1.90)	0.53 (0.50-0.55)	1.09 (1.05-1.13)	1.31 (1.26-1.36)
3	0.52 (0.48-0.57)	1.44 (1.37-1.51)	2.00 (1.92-2.10)	0.52 (0.49-0.56)	1.10 (1.05-1.14)	1.27 (1.23-1.33)
4	0.54 (0.50-0.59)	1.36 (1.28-1.44)	2.01 (1.91-2.11)	0.55 (0.52-0.58)	0.97 (0.92-1.01)	1.21 (1.16-1.27)
5	0.53 (0.48-0.58)	1.26 (1.18-1.34)	2.04 (1.93-2.16)	0.54 (0.51-0.58)	0.96 (0.91-1.01)	1.20 (1.14-1.25)
6	0.56 (0.51-0.62)	1.28 (1.19-1.37)	2.10 (1.98-2.23)	0.58 (0.54-0.62)	0.94 (0.89-0.99)	1.16 (1.11-1.22)
7	0.58 (0.52-0.64)	1.20 (1.11-1.30)	2.32 (2.18-2.47)	0.55 (0.51-0.59)	0.95 (0.90-1.00)	1.24 (1.18-1.30)
8	0.54 (0.48-0.61)	1.33 (1.22-1.44)	2.33 (2.18-2.49)	0.54 (0.50-0.58)	1.04 (0.98-1.10)	1.21 (1.15-1.28)
9	0.55 (0.48-0.62)	1.22 (1.12-1.33)	2.19 (2.04-2.34)	0.54 (0.51-0.59)	0.98 (0.92-1.04)	1.15 (1.09-1.21)
10	0.53 (0.47-0.61)	1.27 (1.15-1.39)	2.38 (2.21-2.56)	0.55 (0.51-0.59)	1.06 (1.00-1.13)	1.19 (1.13-1.26)
11	0.52 (0.45-0.60)	1.38 (1.26-1.51)	2.41 (2.23-2.60)	0.52 (0.48-0.56)	1.12 (1.06-1.19)	1.21 (1.14-1.28)
Age group, mo						
0-5	0.52 (0.51-0.54)	1.27 (1.25-1.30)	1.77 (1.74-1.80)	0.53 (0.52-0.54)	1.05 (1.04-1.07)	1.26 (1.24-1.28)
6-11	0.55 (0.52-0.58)	1.27 (1.23-1.32)	2.27 (2.21-2.33)	0.55 (0.53-0.57)	1.01 (0.98-1.03)	1.19 (1.17-1.22)
12-23	0.55 (0.52-0.57)	1.52 (1.47-1.57)	3.90 (3.81-3.98)	0.58 (0.56-0.59)	1.39 (1.36-1.41)	1.77 (1.74-1.80)
24-59	0.54 (0.51-0.57)	1.59 (1.54-1.64)	4.86 (4.75-4.98)	0.59 (0.57-0.62)	1.56 (1.52-1.60)	3.19 (3.13-3.26)

Abbreviation: RSV, respiratory syncytial virus.

^a^ Overall hospital admissions (intensive care unit and inpatient).

With few exceptions, there was an increased incidence of RSV and a decreased incidence of non-RSV bronchiolitis encounters across age groups and care units in calendar years 2021 and 2022 compared with 2015 to 2019 (Table 3; eTables 1 and 2 in Supplement 1). Between 2015 and 2019, 72 428 of 425 433 patients with non-RSV bronchiolitis (17.0%) had RSV testing, compared with 37 535 of 150 019 (25.0%) between 2020 and 2022.

Among older children with RSV in 2022, those aged 12 to 23 months were 3.90 times as likely (95% CI, 3.81-3.98) to have a hospital encounter compared with 2015 to 2019, whereas those aged 24 to 59 months of age were 4.86 times as likely (95% CI, 4.75-4.98) and infants aged 0 to 5 months were 1.77 (95% CI, 1.74-1.80) times as likely (Table 3). Incidences (Table 2) and IRRs (Table 3; eTables 1 and 2 in Supplement 1) by age group and care unit are presented in their respective tables.

Hospitalization IRRs by Medicaid are provided in eTable 3 in Supplement 1. Overall, boys were more likely to be hospitalized than girls, with IRRs ranging from 1.12 (95% CI, 1.08-1.16) to 1.53 (95% CI, 1.46-1.59) for all years studied. Similarly, Medicaid patients were more likely to be hospitalized or have any patient encounter than non-Medicaid patients (eTable 3 in Supplement 1) across care units. The sex ratios remained similar for all age groups in the prepandemic, pandemic (2020), and postpandemic (2021 and 2022) years. However, although the IRR remained greater than 1 for Medicaid patients with few exceptions, there was a relative decrease in the proportion of Medicaid patients accessing care across units for all 3 years after the pandemic (eTable 4 in Supplement 1).

## Discussion

This study presented a unique opportunity to use a large, nationwide sample to examine distinct patterns in burden of disease between RSV and non-RSV bronchiolitis before and after the COVID-19 pandemic. Additionally, regional distinctions could be made to identify potential for variation among US regions in climate and therefore variations in timing, severity, and duration of RSV season. Significant differences in patient characteristics between cohorts are due to large sample sizes and perhaps not clinically meaningful; however, the proportion of ICU admissions in the RSV (19.7%) relative to the non-RSV bronchiolitis (8.6%) cohort may have more clinical relevance. The PHIS database allows for specification of *ICD* codes, such that RSV and non-RSV bronchiolitis encounters could be examined separately, where these codes are commonly combined. The IRRs for the combined cohort suggest that analysis of health care resource utilization (HCRU) of a combined cohort may not be reflective of either distinct RSV- or non-RSV bronchiolitis–specific HCRU (Table 3; eTables 1 and 2 in Supplement 1).

The current findings suggest that the relative risk of RSV-specific HCRU across age groups increased in postpandemic seasons (2021-2022) relative to before the pandemic (2015-2019). Furthermore, the risk of hospitalization, as well as ED and observation unit admissions, increased in magnitude with age, suggesting a disproportionately increased burden in older children. Infants may have had less exposure to RSV during the COVID-19 pandemic in 2020 to 2021, which may have increased the susceptibility of those 12 months or older during 2021 to 2022. The authors speculate that some of this is due to the increased testing observed among the non-RSV bronchiolitis cohort; the finding that the IRRs in this cohort were generally less than 1 suggests an increase in RSV test positivity. Interestingly, however, the EDs showed lower rates of use in patients up to 23 months of age, whereas the oldest age group (24-59 months) saw a 1.7- to 2.0-fold higher use in 2021 and 2022 (eTable 2 in Supplement 1).

The analysis of Medicaid use suggests a decreased burden among Medicaid compared with commercially insured patients (eTable 3 in Supplement 1). Although this result is paradoxical, it may be due to a greater proportion of commercially insured patients seeking care after the pandemic. This is suggested by the fact that the relative proportion of Medicaid encounters decreased after the pandemic in 2021 to 2022 (eTable 4 in Supplement 1).

An older age and increased number of inpatient admissions but fewer ICU admissions and milder symptoms among patients with acute lower respiratory tract infection occurred during a delayed 2020 to 2021 RSV resurgence in France^2^; this finding is consistent with the current findings for inpatient but not ICU admissions for RSV during 2021. In addition, on the basis of the current data, no resurgence was observed in the US for this age group until mid-2021 (Figure and Table 3). In Israel, no change in age distribution was observed^5^; however, there was a significant increase in median age observed in Australia in mid-2021.^10^ It is possible that at time of writing there was not yet any significant shift toward older ages, given that the Southern Hemisphere experienced a delayed RSV season sooner. In a comparison of 17 European countries based on surveillance data, van Summeren et al^17^ also observed a shift toward older median age of RSV cases in France and Iceland after these countries began experiencing RSV epidemics in 2021.

It has been suggested that COVID-19, RSV, and other circulating respiratory viruses interact in such ways that they may oppose one another, similarly to the 2009 influenza pandemic, during which the subsequent 2 RSV seasons had delayed onset, despite a lack of NPI measures.^17,34,35^ Additionally, studies in the US suggest an association of longer periods of NPI during the pandemic with a larger susceptible population. Increased susceptibility in older children has implications for future outbreaks and prevention efforts, such as prophylaxis and vaccinations.^19^ How this changing susceptibility across global populations may impact future RSV seasons remains to be seen. If pandemic disruption of RSV circulation influences future seasons, this has implications for older children (ie, aged >12 months), who are typically not prioritized for RSV prophylaxis and who may be disproportionately affected. This analysis indicates the greatest increases in hospitalization were among older children with RSV in 2022 compared with prepandemic seasons, with those 1 year or older at admission showing higher IRRs than those aged 0 to 5 months or 6 to 11 months (Table 3).

The literature indicates that boys in the pediatric population, as well as Medicaid recipients, represent a disproportionate burden of disease in terms of RSV HCRU and mortality. Our data suggest that there was no disproportionate increase in the proportion of either demographic related to the pandemic.

We used a novel method for obtaining population-based estimates for the PHIS database. Although the population estimates were an extrapolation of 2015 to 2016 estimates from RSV incidence reported previously,^33^ by anchoring the data to these estimates, the IRRs were most helpful in drawing conclusions about the age shift and the severity of illness in 2021 and 2022.

### Strengths and Limitations

There were several strengths of the analyses for this study. First, the availability of national data allowed for large sample sizes and for regional differentiation of HCRU. The large number of cases allowed for more precise incidence estimates stratified by care unit and age. The completeness of *ICD* code data in the PHIS database allowed for distinction between RSV and bronchiolitis diagnoses, which, as the results demonstrate, could have been differentially impacted by increased testing for RSV after the COVID-19 pandemic. If this is the case, the combined data may provide a more accurate estimate of the probable burden of RSV.

This study also had some limitations. One notable limitation is the assumption of a relatively static population between 2015 and 2022, given the method of obtaining the population denominator. Additionally, this method assumes that the PHIS hospitals included in this analysis observe a comparable frequency of RSV hospital encounters to those sampled in the study by Rha et al.^33^ Similarly, to estimate incidence by care unit for Medicaid recipients compared with those privately insured, the population at risk was considered to be the total estimated population younger than 5 years. Although it is difficult to obtain a reliable estimate of the Medicaid-specific vs commercially insured population at risk, particularly across the US and by region, such an analysis may provide different risk estimates than the current findings. This calculation is another potential explanation for the proportion of the Medicaid burden appearing to decrease after the pandemic.^33^

Other considerations warranting future study include the role of both climate variation and socioeconomic inequities in these epidemiologic shifts, such as the use of predictive modeling for this purpose. Such modeling would be particularly useful because it is uncertain whether a return to prepandemic RSV seasonality will continue in upcoming years.^20^ In Israel, for example, there was an increase in RSV cases among more densely populated, lower-income neighborhoods, which the authors speculate may be associated with timing of NPI measures.^5^ Climate variability, whether among countries or among regions within the US, may be an additional consideration to minimize the severity of future RSV epidemics, as suggested by this and prior findings.

## Conclusions

This study found that the burden of RSV and related non-RSV bronchiolitis HCRU increased in 2021 and almost doubled in 2022 (except for ED use in the non-RSV and combined cohorts, which decreased) after a decrease during the pandemic in 2020. Older children had absolute increases in HCRU and disease severity, regardless of increases in testing in that population. This finding has implications for newly available prevention strategies (eg, monoclonal antibodies), for which older children are not currently eligible.

## Notes

Supplement 1. **eFigure 1.** Seasonality of RSV Hospital Utilization by HHS Region in Children <60 mo, 2015-2022 **eFigure 2.** RSV Plus Non-RSV Bronchiolitis Hospital Utilization in Patients Under 5 Years of Age in United States PHIS Hospitals, 2015-2023 **eTable 1. **Incidence Rate Ratio of Non-RSV Bronchiolitis Hospitalizations and ICU, Inpatient, Emergency Department, and Observation Unit Admissions in Children <60 Months of Age, 2015-2022 **eTable 2. **Incidence Rate Ratio of RSV and Bronchiolitis ICU, Inpatient, Emergency Department, and Observation Unit Admissions in Infants and Children <60 Months of Age, 2015-2022 **eTable 3. **Incidence Rate Ratio of Hospital Utilization by Medicaid in Infants and Children <60 Months of Age, 2015‐2022 **eTable 4. **RSV and Bronchiolitis Hospital, ICU, Inpatient, Emergency Department, and Observation Unit Admissions in Infants and Children <60 Months of Age, Proportion Medicaid, 2015‐2022

Supplement 2.
Data Sharing Statement
