# Supplementary material for: Respiratory Syncytial Virus Hospital-Based Burden of Disease in Children Younger Than 5 Years, 2015-2022
Source: JAMA Netw Open. 2024 Apr 18;7(4):e247125. doi: 10.1001/jamanetworkopen.2024.7125 (PMC12068875; doi:10.1001/jamanetworkopen.2024.7125)
Supplement: Supplement 1. — eFigure 1. Seasonality of RSV Hospital Utilization by HHS Region in Children <60 mo, 2015-2022 eFigure 2. RSV Plus Non-RSV Bronchiolitis Hospital Utilization in Patients Under 5 Years of Age in United States PHIS Hospitals, 2015-2023 eTable 1. Incidence Rate Ratio of Non-RSV Bronchiolitis Hospitalizations and ICU, Inpatient, Emergency Department, and Observation Unit Admissions in Children <60 Months of Age, 2015-2022 eTable 2. Incidence Rate Ratio of RSV and Bronchiolitis ICU, Inpatient, Emergency Department, and Observation Unit Admissions in Infants and Children <60 Months of Age, 2015-2022 eTable 3. Incidence Rate Ratio of Hospital Utilization by Medicaid in Infants and Children <60 Months of Age, 2015‐2022 eTable 4. RSV and Bronchiolitis Hospital, ICU, Inpatient, Emergency Department, and Observation Unit Admissions in Infants and Children <60 Months of Age, Proportion Medicaid, 2015‐2022 [file jamanetwopen-e247125-s001.pdf]

Supplementary Online Content

Suss RJ, Simões EAF. Respiratory syncytial virus hospital-based burden of disease in children younger than 5 years, 2015-2022. *JAMA Netw Open*. 2024;7(4):e247125. doi:10.1001/jamanetworkopen.2024.7125

eFigure 1. Seasonality of RSV Hospital Utilization by HHS Region in Children <60mo, 2015-2022

eFigure 2. RSV Plus Non-RSV Bronchiolitis Hospital Utilization in Patients Under 5 Years of Age in United States PHIS Hospitals, 2015-2023

eTable 1. Incidence Rate Ratio of non-RSV Bronchiolitis Hospitalizations and ICU, Inpatient, Emergency Department, and Observation Unit Admissions in Children <60 Months of Age, 2015-2022

eTable 2. Incidence Rate Ratio of RSV and Bronchiolitis ICU, Inpatient, Emergency Department, and Observation Unit Admissions in Infants and Children <60 Months of Age, 2015-2022

eTable 3. Incidence Rate Ratio of Hospital Utilization by Medicaid in Infants and Children <60 Months of Age, 2015-2022

eTable 4. RSV and Bronchiolitis Hospital, ICU, Inpatient, Emergency Department, and Observation Unit Admissions in Infants and Children <60 Months of Age, Proportion Medicaid, 2015-2022

This supplementary material has been provided by the authors to give readers additional information about their work.

© 2024 Suss RJ et al. *JAMA Network Open.*

eFigure 1. Seasonality of RSV Hospital Utilization by HHS Region in Children <60mo, 2015-2022.

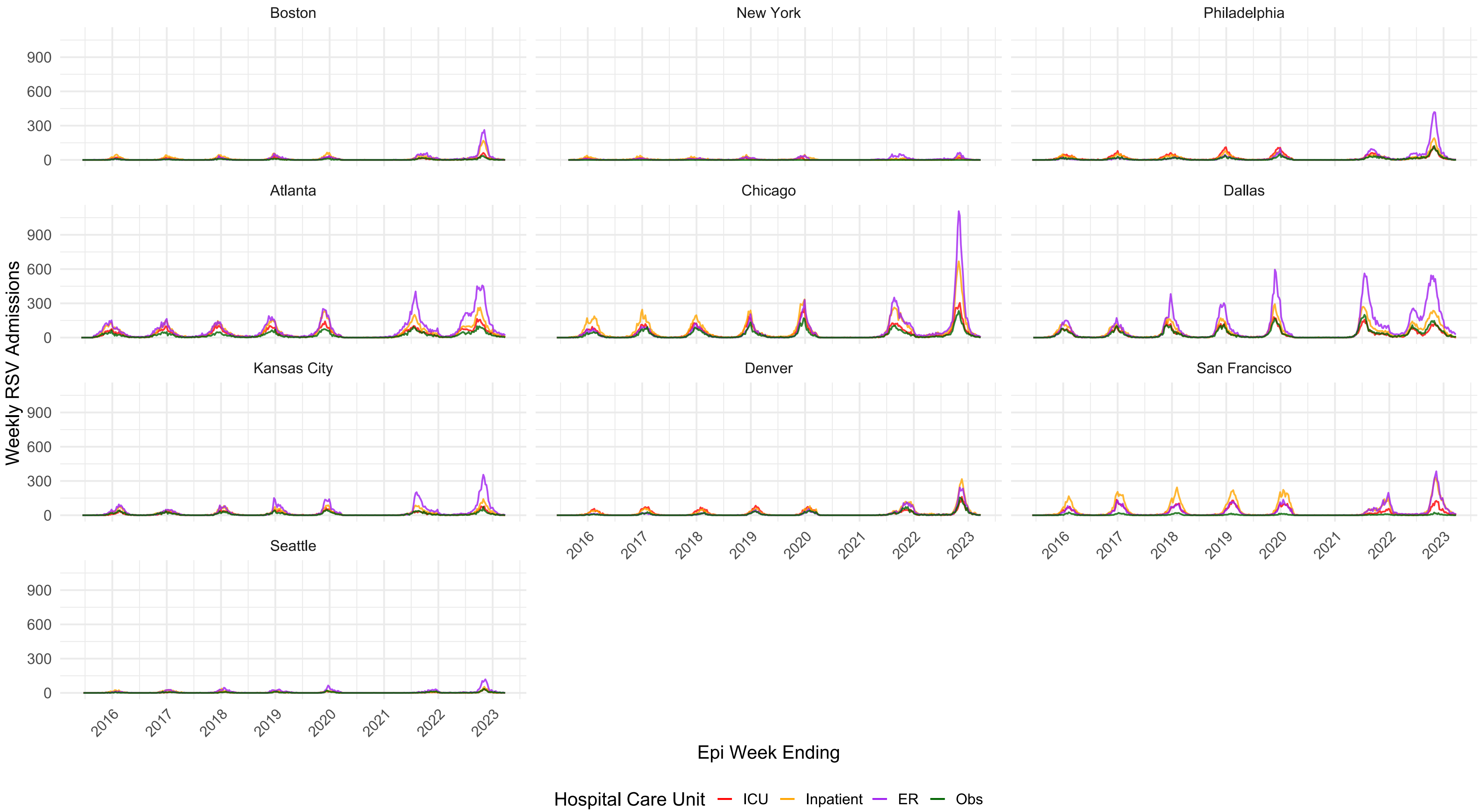

eFigure 1. Total weekly admissions per epi-year by unit of care: ICU, Inpatient, Emergency Department, or Observation Unit. Counts reflect regional totals for all PHIS hospitals on the epi-week ending calendar date, for RSV seasons starting 2015-2022. Weekly totals in part reflect the number of reporting hospitals within each region.

© 2024 Suss RJ et al. *JAMA Network Open.*

eFigure 2. RSV Plus Non-RSV Bronchiolitis Hospital Utilization in Patients Under 5 Years of Age in United States PHIS Hospitals, 2015-2023.

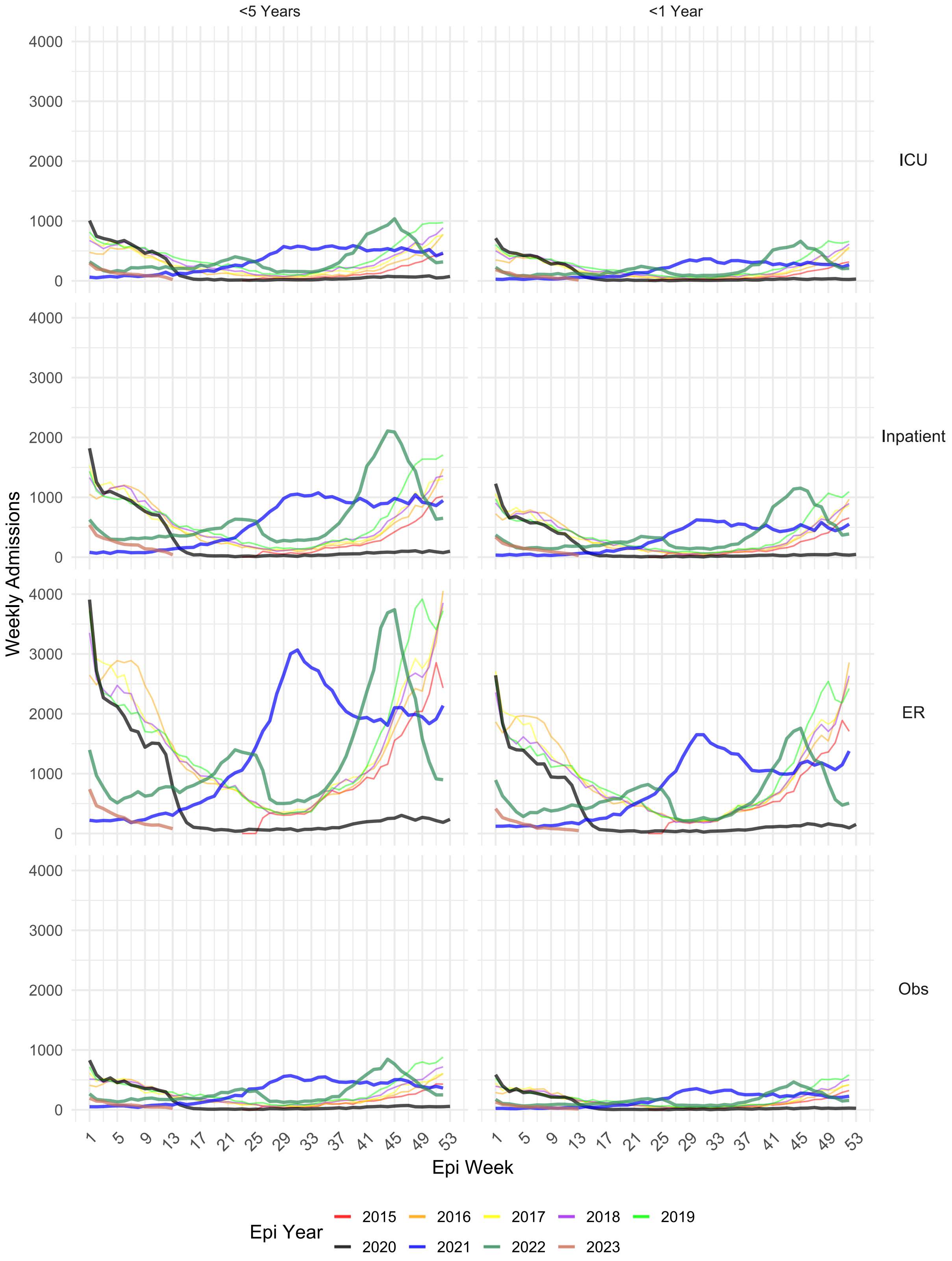

eFigure 2. Seasonality of combined RSV+non-RSV bronchiolitis hospital utilization, epi years 2015-2023 Quarter 1, for patients <5 years (left) and <1 year (right) of age at encounter. Post-pandemic years in bold. Top to bottom: ICU Admissions, Inpatient Admissions, Emergency Admissions, Observation Unit Admissions.

© 2024 Suss RJ et al. *JAMA Network Open.*

eTable 1. Incidence Rate Ratio of non-RSV Bronchiolitis Hospitalizations and ICU, Inpatient, Emergency Department, and Observation Unit Admissions in Children <60 Months of Age, 2015-2022

1A. Incidence Rate Ratio of non-RSV Bronchiolitis Hospitalizations in Infants and Children <60 Months of Age, 2015-2022[a]

Age (months)	IRR (95% CI)					
	2020		2021		2022	
<1	0.54	(0.49-0.61)	0.83	(0.74-0.88)	0.52	(1.12-1.30)
1	0.56	(0.27-0.31)	0.74	(0.15-0.18)	0.46	(0.19-0.21)
2	0.51	(0.32-0.38)	0.73	(0.17-0.20)	0.44	(0.22-0.25)
3	0.52	(0.41-0.48)	0.69	(0.22-0.26)	0.41	(0.27-0.31)
4	0.55	(0.50-0.59)	0.60	(0.28-0.33)	0.46	(0.29-0.34)
5	0.55	(0.56-0.66)	0.71	(0.31-0.36)	0.48	(0.39-0.44)
6	0.59	(0.65-0.76)	0.69	(0.38-0.45)	0.51	(0.44-0.50)
7	0.53	(0.64-0.77)	0.79	(0.35-0.41)	0.55	(0.51-0.58)
8	0.54	(0.68-0.82)	0.87	(0.38-0.44)	0.55	(0.59-0.67)
9	0.54	(0.74-0.89)	0.84	(0.41-0.48)	0.54	(0.62-0.71)
10	0.56	(0.75-0.91)	0.95	(0.43-0.50)	0.57	(0.72-0.82)
11	0.52	(0.74-0.90)	0.99	(0.40-0.46)	0.58	(0.73-0.84)
Age Groups						
0-5	0.54	(0.36-0.39)	0.71	(0.20-0.21)	0.46	(0.25-0.26)
6-11	0.55	(0.73-0.79)	0.85	(0.41-0.43)	0.55	(0.61-0.64)
12-23	0.60	(0.48-0.51)	1.32	(0.29-0.31)	0.64	(0.62-0.65)
24-59	0.67	(0.14-0.16)	1.53	(0.10-0.11)	0.84	(0.22-0.23)

a. Overall hospital admissions (ICU and inpatient)

© 2024 Suss RJ et al. *JAMA Network Open.*

1B. Incidence Rate Ratio of non-RSV Bronchiolitis ICU Admissions in Infants and Children <60 Months of Age, 2015-2022

Age (months)	IRR (95% CI)					
	2020		2021		2022	
<1	0.67	(0.57-0.78)	0.84	(0.73-0.97)	0.55	(0.46-0.65)
1	0.20	(0.18-0.23)	0.13	(0.12-0.15)	0.17	(0.15-0.19)
2	0.25	(0.22-0.29)	0.15	(0.13-0.17)	0.21	(0.18-0.24)
3	0.34	(0.30-0.39)	0.23	(0.20-0.26)	0.30	(0.26-0.35)
4	0.49	(0.42-0.56)	0.33	(0.29-0.38)	0.35	(0.30-0.41)
5	0.53	(0.46-0.62)	0.33	(0.29-0.38)	0.41	(0.35-0.49)
6	0.68	(0.59-0.79)	0.48	(0.42-0.55)	0.58	(0.50-0.67)
7	0.66	(0.57-0.77)	0.47	(0.41-0.54)	0.64	(0.55-0.75)
8	0.75	(0.65-0.88)	0.50	(0.45-0.57)	0.86	(0.73-1.01)
9	0.90	(0.78-1.05)	0.60	(0.53-0.68)	0.96	(0.82-1.12)
10	0.95	(0.81-1.12)	0.57	(0.50-0.64)	1.09	(0.94-1.27)
11	0.87	(0.74-1.03)	0.52	(0.46-0.59)	1.01	(0.86-1.20)
Age Groups						
0-5	0.28	(0.27-0.30)	0.18	(0.17-0.19)	0.23	(0.22-0.25)
6-11	0.79	(0.74-0.84)	0.52	(0.49-0.55)	0.83	(0.78-0.88)
12-23	0.89	(0.84-0.94)	0.62	(0.60-0.64)	1.49	(1.41-1.58)
24-59	0.29	(0.27-0.32)	0.23	(0.21-0.24)	0.50	(0.46-0.54)

© 2024 Suss RJ et al. *JAMA Network Open.*

1C. Incidence Rate Ratio of non-RSV Bronchiolitis Inpatient Admissions in Infants and Children <60 Months of Age, 2015-2022

Age (months)	IRR (95% CI)					
	2020		2021		2022	
<1	0.46	(0.39-0.53)	0.83	(0.74-0.93)	0.50	(0.43-0.57)
1	0.34	(0.31-0.37)	0.18	(0.16-0.19)	0.24	(0.21-0.26)
2	0.40	(0.36-0.45)	0.19	(0.17-0.21)	0.27	(0.24-0.31)
3	0.48	(0.43-0.54)	0.23	(0.20-0.25)	0.29	(0.26-0.33)
4	0.57	(0.52-0.63)	0.29	(0.26-0.32)	0.31	(0.28-0.35)
5	0.64	(0.58-0.71)	0.34	(0.31-0.37)	0.43	(0.39-0.48)
6	0.71	(0.64-0.79)	0.39	(0.35-0.43)	0.45	(0.40-0.51)
7	0.72	(0.64-0.81)	0.33	(0.30-0.36)	0.52	(0.47-0.58)
8	0.75	(0.67-0.84)	0.36	(0.33-0.40)	0.56	(0.50-0.62)
9	0.79	(0.70-0.89)	0.38	(0.35-0.42)	0.59	(0.52-0.66)
10	0.80	(0.71-0.90)	0.43	(0.39-0.47)	0.68	(0.61-0.77)
11	0.80	(0.71-0.91)	0.39	(0.35-0.42)	0.72	(0.64-0.81)
Age Groups						
0-5	0.42	(0.40-0.44)	0.21	(0.20-0.22)	0.28	(0.27-0.29)
6-11	0.76	(0.72-0.79)	0.38	(0.36-0.39)	0.57	(0.55-0.60)
12-23	0.79	(0.76-0.83)	0.43	(0.42-0.45)	0.91	(0.87-0.95)
24-59	0.19	(0.18-0.21)	0.12	(0.11-0.12)	0.28	(0.26-0.30)

© 2024 Suss RJ et al. *JAMA Network Open.*

1D. Incidence Rate Ratio of non-RSV Bronchiolitis Emergency Department Admissions in Infants and Children <60 Months of Age, 2015-2022

Age (months)	IRR (95% CI)					
	2020		2021		2022	
<1	0.46	(0.41-0.53)	0.93	(0.85-1.03)	0.30	(0.26-0.36)
1	0.43	(0.41-0.46)	0.84	(0.80-0.89)	0.35	(0.32-0.38)
2	0.39	(0.36-0.41)	0.75	(0.71-0.78)	0.29	(0.27-0.31)
3	0.42	(0.40-0.44)	0.74	(0.71-0.77)	0.29	(0.27-0.31)
4	0.42	(0.40-0.44)	0.71	(0.69-0.74)	0.28	(0.27-0.30)
5	0.41	(0.39-0.43)	0.69	(0.66-0.71)	0.31	(0.29-0.32)
6	0.41	(0.39-0.43)	0.70	(0.68-0.73)	0.30	(0.29-0.32)
7	0.41	(0.39-0.43)	0.69	(0.66-0.72)	0.30	(0.29-0.32)
8	0.40	(0.37-0.42)	0.73	(0.70-0.77)	0.30	(0.28-0.32)
9	0.38	(0.36-0.41)	0.73	(0.69-0.76)	0.32	(0.30-0.34)
10	0.42	(0.39-0.44)	0.75	(0.71-0.79)	0.30	(0.28-0.33)
11	0.38	(0.35-0.40)	0.82	(0.78-0.86)	0.29	(0.26-0.31)
Age Groups						
0-5	0.42	(0.41-0.43)	0.74	(0.73-0.75)	0.30	(0.29-0.31)
6-11	0.40	(0.39-0.41)	0.73	(0.72-0.74)	0.30	(0.30-0.31)
12-23	0.43	(0.42-0.44)	0.92	(0.91-0.94)	0.33	(0.32-0.34)
24-59	0.33	(0.31-0.35)	0.93	(0.90-0.96)	0.32	(0.30-0.33)

© 2024 Suss RJ et al. *JAMA Network Open.*

1E. Incidence Rate Ratio of non-RSV Bronchiolitis Observation Unit Admissions in Infants and Children <60 Months of Age, 2015-2022

Age (months)	IRR (95% CI)					
	2020		2021		2022	
<1	0.61	(0.51-0.74)	1.29	(1.12-1.48)	0.47	(0.38-0.59)
1	0.56	(0.49-0.63)	0.90	(0.81-0.99)	0.44	(0.38-0.50)
2	0.49	(0.43-0.56)	0.83	(0.75-0.92)	0.36	(0.31-0.42)
3	0.59	(0.52-0.67)	0.74	(0.66-0.83)	0.32	(0.27-0.38)
4	0.57	(0.50-0.65)	0.75	(0.67-0.84)	0.34	(0.29-0.40)
5	0.60	(0.53-0.68)	0.68	(0.61-0.77)	0.39	(0.34-0.46)
6	0.56	(0.49-0.64)	0.76	(0.68-0.86)	0.51	(0.44-0.58)
7	0.60	(0.53-0.69)	0.81	(0.72-0.92)	0.45	(0.38-0.52)
8	0.51	(0.44-0.59)	0.89	(0.79-1.0)	0.43	(0.36-0.51)
9	0.48	(0.41-0.57)	0.74	(0.65-0.84)	0.47	(0.40-0.55)
10	0.54	(0.46-0.64)	0.86	(0.76-0.98)	0.47	(0.39-0.55)
11	0.53	(0.45-0.62)	0.85	(0.74-0.97)	0.46	(0.39-0.54)
Age Groups						
0-5	0.56	(0.54-0.60)	0.82	(0.78-0.86)	0.38	(0.35-0.40)
6-11	0.54	(0.51-0.57)	0.82	(0.78-0.86)	0.46	(0.43-0.50)
12-23	0.60	(0.57-0.64)	1.16	(1.11-1.21)	0.58	(0.55-0.61)
24-59	0.62	(0.55-0.69)	1.58	(1.47-1.70)	0.75	(0.68-0.83)

© 2024 Suss RJ et al. *JAMA Network Open.*

eTable 2. Incidence Rate Ratio of RSV and Bronchiolitis ICU, Inpatient, Emergency Department, and Observation Unit Admissions in Infants and Children <60 Months of Age, 2015-2022

2A. Incidence Rate Ratio of RSV and Bronchiolitis ICU Admissions in Infants and Children <60 Months of Age, 2015-2022

Age (months)	RSV IRR (95% CI) 2020		2021		2022		RSV+Bronchiolitis IRR (95% CI) 2020		2021		2022	
<1	0.50	(0.47-0.53)	1.18	(1.13-1.23)	1.51	(1.45-1.57)	0.51	(0.48-0.54)	1.10	(1.05-1.14)	1.27	(1.22-1.32)
1	0.52	(0.49-0.55)	1.21	(1.16-1.26)	1.64	(1.58-1.69)	0.53	(0.51-0.56)	1.07	(1.03-1.10)	1.28	(1.24-1.32)
2	0.53	(0.50-0.57)	1.31	(1.25-1.37)	1.83	(1.76-1.90)	0.53	(0.50-0.55)	1.09	(1.05-1.13)	1.31	(1.26-1.36)
3	0.52	(0.48-0.57)	1.44	(1.37-1.51)	2.00	(1.92-2.10)	0.52	(0.49-0.56)	1.10	(1.05-1.14)	1.27	(1.23-1.33)
4	0.54	(0.50-0.59)	1.36	(1.28-1.44)	2.01	(1.91-2.11)	0.55	(0.52-0.58)	0.97	(0.92-1.01)	1.21	(1.16-1.27)
5	0.53	(0.48-0.58)	1.26	(1.18-1.34)	2.04	(1.93-2.16)	0.54	(0.51-0.58)	0.96	(0.91-1.01)	1.20	(1.14-1.25)
6	0.56	(0.51-0.62)	1.28	(1.19-1.37)	2.10	(1.98-2.23)	0.58	(0.54-0.62)	0.94	(0.89-0.99)	1.16	(1.11-1.22)
7	0.58	(0.52-0.64)	1.20	(1.11-1.30)	2.32	(2.18-2.47)	0.55	(0.51-0.59)	0.95	(0.90-1.0)	1.24	(1.18-1.30)
8	0.54	(0.48-0.61)	1.33	(1.22-1.44)	2.33	(2.18-2.49)	0.54	(0.50-0.58)	1.04	(0.98-1.10)	1.21	(1.15-1.28)
9	0.55	(0.48-0.62)	1.22	(1.12-1.33)	2.19	(2.04-2.34)	0.54	(0.51-0.59)	0.98	(0.92-1.04)	1.15	(1.09-1.21)
10	0.53	(0.47-0.61)	1.27	(1.15-1.39)	2.38	(2.21-2.56)	0.55	(0.51-0.59)	1.06	(1.00-1.13)	1.19	(1.13-1.26)
11	0.52	(0.45-0.60)	1.38	(1.26-1.51)	2.41	(2.23-2.60)	0.52	(0.48-0.56)	1.12	(1.06-1.19)	1.21	(1.14-1.28)
**Age Groups**												
0-5	0.53	(.51-.55)	1.14	(1.10-1.17)	1.52	(1.48-1.56)	0.57	(0.55-0.59)	1.04	(1.01-1.07)	1.23	(1.20-1.26)
6-11	0.54	(.50-.59)	1.08	(1.02-1.15)	1.74	(1.65-1.82)	0.61	(0.58-0.64)	1.06	(1.02-1.11)	1.10	(1.06-1.15)
12-23	0.53	(.49-.58)	1.25	(1.19-1.33)	1.84	(1.76-1.93)	0.63	(0.61-0.66)	1.52	(1.47-1.56)	1.14	(1.10-1.18)
24-59	0.52	(.47-.56)	1.32	(1.24-1.40)	2.56	(2.44-2.68)	0.63	(0.59-0.67)	1.49	(1.42-1.55)	1.86	(1.79-1.93)

© 2024 Suss RJ et al. *JAMA Network Open.*

2B. Incidence Rate Ratio of RSV and Bronchiolitis Inpatient Admissions in Infants and Children <60 Months of Age, 2015-2022

	RSV						RSV+Bronchiolitis					
	IRR (95% CI)						IRR (95% CI)					
**Age (months)**	2020		2021		2022		2020		2021		2022	
<1	0.49	(0.44-0.53)	1.28	(1.20-1.35)	1.51	(1.43-1.59)	0.48	(0.44-0.52)	1.16	(1.10-1.22)	1.24	(1.18-1.30)
1	0.48	(0.45-0.52)	1.27	(1.20-1.33)	1.70	(1.63-1.78)	0.50	(0.47-0.53)	1.06	(1.01-1.11)	1.22	(1.17-1.27)
2	0.55	(0.50-0.60)	1.43	(1.35-1.52)	1.99	(1.89-2.09)	0.51	(0.48-0.55)	1.10	(1.05-1.15)	1.28	(1.22-1.34)
3	0.54	(0.48-0.60)	1.63	(1.53-1.74)	2.20	(2.07-2.33)	0.50	(0.46-0.54)	1.10	(1.04-1.16)	1.24	(1.18-1.31)
4	0.55	(0.49-0.61)	1.50	(1.39-1.61)	2.21	(2.08-2.36)	0.52	(0.48-0.56)	0.97	(0.91-1.02)	1.21	(1.15-1.28)
5	0.52	(0.46-0.58)	1.38	(1.28-1.50)	2.21	(2.07-2.37)	0.52	(0.48-0.56)	0.97	(0.91-1.03)	1.18	(1.12-1.25)
6	0.58	(0.51-0.66)	1.40	(1.29-1.53)	2.31	(2.15-2.48)	0.56	(0.52-0.60)	0.92	(0.87-0.99)	1.15	(1.08-1.22)
7	0.57	(0.50-0.66)	1.34	(1.21-1.48)	2.53	(2.34-2.74)	0.49	(0.45-0.54)	0.94	(0.88-1.00)	1.22	(1.15-1.30)
8	0.57	(0.49-0.66)	1.45	(1.31-1.60)	2.47	(2.28-2.69)	0.52	(0.47-0.56)	0.99	(0.92-1.06)	1.22	(1.14-1.30)
9	0.52	(0.45-0.61)	1.29	(1.16-1.43)	2.38	(2.18-2.59)	0.50	(0.45-0.55)	0.93	(0.87-1.00)	1.16	(1.08-1.24)
10	0.52	(0.44-0.62)	1.35	(1.21-1.52)	2.55	(2.33-2.79)	0.53	(0.48-0.59)	1.02	(0.95-1.10)	1.18	(1.10-1.27)
11	0.55	(0.47-0.66)	1.55	(1.38-1.73)	2.57	(2.34-2.82)	0.50	(0.45-0.56)	1.11	(1.03-1.19)	1.23	(1.15-1.32)
**Age Groups**												
0-5	0.51	(0.49-0.53)	1.38	(1.35-1.42)	1.89	(1.85-1.93)	0.50	(0.49-0.52)	1.06	(1.04-1.09)	1.23	(1.21-1.26)
6-11	0.56	(0.52-0.59)	1.39	(1.33-1.45)	2.45	(2.37-2.54)	0.52	(0.50-0.54)	0.98	(0.95-1.01)	1.19	(1.16-1.22)
12-23	0.55	(0.52-0.59)	1.68	(1.61-1.74)	2.56	(2.48-2.65)	0.55	(0.53-0.57)	1.32	(1.29-1.35)	1.25	(1.22-1.29)
24-59	0.55	(0.52-0.59)	1.73	(1.67-1.80)	3.58	(3.47-3.69)	0.57	(0.54-0.60)	1.61	(1.56-1.66)	2.44	(2.37-2.51)

© 2024 Suss RJ et al. *JAMA Network Open.*

2C. Incidence Rate Ratio of RSV and Bronchiolitis Emergency Department Admissions in Infants and Children <60 Months of Age, 2015-2022

	RSV						RSV+Bronchiolitis					
	IRR (95% CI)						IRR (95% CI)					
**Age (months)**	2020		2021		2022		2020		2021		2022	
<1	0.39	(0.32-0.48)	2.03	(1.83-2.24)	2.95	(2.70-3.22)	0.44	(0.39-0.49)	1.31	(1.23-1.41)	1.23	(1.14-1.32)
1	0.47	(0.42-0.52)	1.62	(1.52-1.72)	2.67	(2.54-2.81)	0.44	(0.42-0.47)	1.06	(1.02-1.10)	1.01	(0.97-1.05)
2	0.50	(0.45-0.55)	1.61	(1.52-1.71)	2.72	(2.59-2.86)	0.41	(0.39-0.43)	0.93	(0.90-0.96)	0.80	(0.77-0.83)
3	0.52	(0.46-0.57)	1.78	(1.68-1.90)	3.19	(3.03-3.36)	0.44	(0.42-0.46)	0.91	(0.88-0.94)	0.75	(0.72-0.78)
4	0.55	(0.50-0.62)	2.00	(1.88-2.13)	3.29	(3.12-3.47)	0.44	(0.42-0.46)	0.89	(0.86-0.91)	0.69	(0.66-0.71)
5	0.59	(0.53-0.66)	1.93	(1.81-2.07)	3.53	(3.34-3.73)	0.43	(0.41-0.45)	0.84	(0.81-0.86)	0.69	(0.67-0.72)
6	0.54	(0.48-0.61)	1.94	(1.80-2.08)	3.63	(3.43-3.85)	0.42	(0.40-0.44)	0.85	(0.82-0.88)	0.69	(0.67-0.72)
7	0.58	(0.51-0.66)	2.23	(2.06-2.40)	4.08	(3.83-4.34)	0.43	(0.41-0.45)	0.85	(0.82-0.88)	0.71	(0.68-0.73)
8	0.50	(0.43-0.58)	2.03	(1.87-2.21)	4.04	(3.78-4.32)	0.41	(0.39-0.43)	0.88	(0.85-0.91)	0.72	(0.69-0.75)
9	0.56	(0.49-0.65)	2.21	(2.03-2.40)	4.21	(3.93-4.52)	0.40	(0.38-0.43)	0.89	(0.86-0.93)	0.75	(0.72-0.79)
10	0.54	(0.46-0.63)	2.19	(2.0-2.39)	4.43	(4.12-4.76)	0.43	(0.40-0.46)	0.92	(0.88-0.96)	0.78	(0.75-0.82)
11	0.58	(0.49-0.68)	2.43	(2.22-2.66)	4.39	(4.07-4.73)	0.40	(0.37-0.43)	1.01	(0.96-1.05)	0.77	(0.73-0.81)
**Age Groups**												
0-5	0.51	(0.49-0.54)	1.79	(1.74-1.84)	3.03	(2.96-3.10)	0.43	(0.42-0.44)	0.92	(0.91-0.94)	0.78	(0.77-0.79)
6-11	0.55	(0.52-0.58)	2.14	(2.07-2.21)	4.07	(3.96-4.18)	0.42	(0.41-0.43)	0.89	(0.88-0.90)	0.73	(0.72-0.74)
12-23	0.67	(0.64-0.71)	3.23	(3.13-3.33)	5.37	(5.22-5.51)	0.46	(0.45-0.47)	1.21	(1.19-1.23)	0.96	(0.94-0.97)
24-59	0.76	(0.71-0.81)	5.32	(5.14-5.50)	10.36	(10.07-10.67)	0.41	(0.39-0.42)	1.73	(1.70-1.77)	2.16	(2.12-2.20)

© 2024 Suss RJ et al. *JAMA Network Open.*

2D. Incidence Rate Ratio of RSV and Bronchiolitis Observation Unit Admissions in Infants and Children <60 Months of Age, 2015-2022

	RSV IRR (95% CI)						RSV+Bronchiolitis IRR (95% CI)					
Age (months)	2020		2021		2022		2020		2021		2022	
<1	0.39	(0.32-0.48)	2.03	(1.83-2.24)	2.95	(2.70-3.22)	0.44	(0.39-0.49)	1.31	(1.23-1.41)	1.23	(1.14-1.32)
1	0.47	(0.42-0.52)	1.62	(1.52-1.72)	2.67	(2.54-2.81)	0.44	(0.42-0.47)	1.06	(1.02-1.10)	1.01	(0.97-1.05)
2	0.50	(0.45-0.55)	1.61	(1.52-1.71)	2.72	(2.59-2.86)	0.41	(0.39-0.43)	0.93	(0.90-0.96)	0.80	(0.77-0.83)
3	0.52	(0.46-0.57)	1.78	(1.68-1.90)	3.19	(3.03-3.36)	0.44	(0.42-0.46)	0.91	(0.88-0.94)	0.75	(0.72-0.78)
4	0.55	(0.50-0.62)	2.00	(1.88-2.13)	3.29	(3.12-3.47)	0.44	(0.42-0.46)	0.89	(0.86-0.91)	0.69	(0.66-0.71)
5	0.59	(0.53-0.66)	1.93	(1.81-2.07)	3.53	(3.34-3.73)	0.43	(0.41-0.45)	0.84	(0.81-0.86)	0.69	(0.67-0.72)
6	0.54	(0.48-0.61)	1.94	(1.80-2.08)	3.63	(3.43-3.85)	0.42	(0.40-0.44)	0.85	(0.82-0.88)	0.69	(0.67-0.72)
7	0.58	(0.51-0.66)	2.23	(2.06-2.40)	4.08	(3.83-4.34)	0.43	(0.41-0.45)	0.85	(0.82-0.88)	0.71	(0.68-0.73)
8	0.50	(0.43-0.58)	2.03	(1.87-2.21)	4.04	(3.78-4.32)	0.41	(0.39-0.43)	0.88	(0.85-0.91)	0.72	(0.69-0.75)
9	0.56	(0.49-0.65)	2.21	(2.03-2.40)	4.21	(3.93-4.52)	0.40	(0.38-0.43)	0.89	(0.86-0.93)	0.75	(0.72-0.79)
10	0.54	(0.46-0.63)	2.19	(2.00-2.39)	4.43	(4.12-4.76)	0.43	(0.40-0.46)	0.92	(0.88-0.96)	0.78	(0.75-0.82)
11	0.58	(0.49-0.68)	2.43	(2.22-2.66)	4.39	(4.07-4.73)	0.40	(0.37-0.43)	1.01	(0.96-1.05)	0.77	(0.73-0.81)
**Age Groups**												
0-5	0.51	(0.49-0.54)	1.79	(1.74-1.84)	3.03	(2.96-3.10)	0.43	(0.42-0.44)	0.92	(0.91-0.94)	0.78	(0.77-0.79)
6-11	0.55	(0.52-0.58)	2.14	(2.07-2.21)	4.07	(3.96-4.18)	0.42	(0.41-0.43)	0.89	(0.88-0.90)	0.73	(0.72-0.74)
12-23	0.67	(0.64-0.71)	3.23	(3.13-3.33)	5.37	(5.22-5.51)	0.46	(0.45-0.47)	1.21	(1.19-1.23)	0.96	(0.94-0.97)
24-59	0.76	(0.71-0.81)	5.32	(5.14-5.50)	10.36	(10.07-10.67)	0.41	(0.39-0.42)	1.73	(1.70-1.77)	2.16	(2.12-2.20)

© 2024 Suss RJ et al. *JAMA Network Open.*

eTable 3. Incidence Rate Ratio of Hospital Utilization by Medicaid in Infants and Children <60 Months of Age, 2015-2022[a]

		RSV				Bronchiolitis Only				RSV+Bronchiolitis			
Age (months)		IRR (95% CI)				IRR (95% CI)				IRR (95% CI)			
		2015-2019	2020	2021	2022	2015-2019	2020	2021	2022	2015-2019	2020	2021	2022
Hospitalized[a]	0-5	1.57 (1.54, 1.59)	1.38 (1.31, 1.46)	1.23 (1.19, 1.27)	1.10 (1.07, 1.13)	1.89 (1.85, 1.94)	1.83 (1.70, 1.96)	1.60 (1.50, 1.70)	1.53 (1.42, 1.65)	1.68 (1.66, 1.71)	1.54 (1.48, 1.61)	1.31 (1.28, 1.35)	1.16 (1.12, 1.19)
	6-11	1.55 (1.50, 1.60)	1.38 (1.26, 1.52)	1.31 (1.23, 1.39)	1.10 (1.05, 1.15)	1.90 (1.85, 1.95)	1.75 (1.62, 1.89)	1.73 (1.63, 1.84)	1.39 (1.30, 1.49)	1.76 (1.73, 1.79)	1.60 (1.51, 1.70)	1.51 (1.45, 1.58)	1.18 (1.14, 1.23)
	12-23	1.36 (1.32, 1.40)	1.25 (1.14, 1.38)	1.28 (1.21, 1.35)	1.06 (1.01, 1.11)	1.41 (1.38, 1.44)	1.33 (1.25, 1.42)	1.31 (1.26, 1.37)	1.33 (1.26, 1.42)	1.39 (1.37, 1.42)	1.31 (1.24, 1.38)	1.30 (1.26, 1.34)	1.15 (1.11, 1.19)
	24-59	1.27 (1.23, 1.31)	1.19 (1.07, 1.31)	1.23 (1.16, 1.30)	1.04 (1.00, 1.08)	1.41 (1.35, 1.46)	1.50 (1.35, 1.67)	1.23 (1.15, 1.32)	1.26 (1.15, 1.38)	1.32 (1.29, 1.36)	1.32 (1.23, 1.42)	1.23 (1.17, 1.28)	1.07 (1.03, 1.11)
ICU	0-5	1.63 (1.58, 1.67)	1.41 (1.30, 1.53)	1.32 (1.25, 1.40)	1.20 (1.14, 1.25)	2.12 (2.03, 2.21)	1.98 (1.76, 2.23)	1.91 (1.72, 2.11)	1.72 (1.52, 1.94)	1.76 (1.72, 1.80)	1.58 (1.48, 1.69)	1.44 (1.37, 1.51)	1.26 (1.21, 1.32)
	6-11	1.57 (1.49, 1.65)	1.23 (1.06, 1.43)	1.42 (1.27, 1.58)	1.15 (1.06, 1.25)	2.01 (1.92, 2.10)	2.00 (1.77, 2.27)	2.06 (1.86, 2.27)	1.67 (1.48, 1.88)	1.81 (1.75, 1.87)	1.66 (1.51, 1.83)	1.74 (1.62, 1.88)	1.29 (1.21, 1.38)
	12-23	1.32 (1.26, 1.39)	1.30 (1.11, 1.51)	1.37 (1.24, 1.51)	1.08 (1.0, 1.17)	1.51 (1.45, 1.57)	1.31 (1.18, 1.45)	1.45 (1.35, 1.55)	1.48 (1.34, 1.64)	1.43 (1.39, 1.48)	1.30 (1.19, 1.42)	1.42 (1.35, 1.51)	1.23 (1.16, 1.31)
	24-59	1.33 (1.26, 1.41)	1.18 (.99, 1.40)	1.35 (1.21, 1.50)	1.05 (.98, 1.14)	1.50 (1.41, 1.60)	1.61 (1.37, 1.90)	1.36 (1.22, 1.51)	1.30 (1.12, 1.50)	1.40 (1.34, 1.46)	1.39 (1.23, 1.56)	1.35 (1.25, 1.46)	1.15 (1.07, 1.22)
Inpatient	0-5	1.52 (1.48, 1.56)	1.36 (1.26, 1.47)	1.17 (1.12, 1.23)	1.04 (1.00, 1.08)	1.80 (1.75, 1.85)	1.74 (1.60, 1.91)	1.45 (1.35, 1.56)	1.72 (1.57, 1.89)	1.64 (1.61, 1.67)	1.51 (1.43, 1.60)	1.24 (1.19, 1.29)	1.09 (1.05, 1.13)
	6-11	1.54 (1.48, 1.60)	1.48 (1.31, 1.67)	1.26 (1.17, 1.35)	1.08 (1.02, 1.14)	1.86 (1.80, 1.91)	1.62 (1.48, 1.78)	1.56 (1.45, 1.68)	1.67 (1.52, 1.82)	1.74 (1.70, 1.78)	1.57 (1.46, 1.69)	1.40 (1.33, 1.48)	1.13 (1.08, 1.19)
	12-23	1.38 (1.33, 1.44)	1.23 (1.10, 1.38)	1.24 (1.16, 1.33)	1.05 (1.00, 1.11)	1.37 (1.33, 1.40)	1.35 (1.25, 1.46)	1.23 (1.16, 1.30)	1.48 (1.38, 1.60)	1.37 (1.34, 1.40)	1.31 (1.23, 1.40)	1.23 (1.18, 1.29)	1.11 (1.06, 1.16)
	24-59	1.23 (1.18, 1.28)	1.19 (1.05, 1.34)	1.18 (1.10, 1.26)	1.03 (.98, 1.08)	1.35 (1.29, 1.42)	1.42 (1.23, 1.64)	1.15 (1.05, 1.26)	1.30 (1.15, 1.46)	1.28 (1.24, 1.32)	1.28 (1.17, 1.41)	1.17 (1.10, 1.23)	1.04 (.99, 1.09)
Emergency	0-5	1.65 (1.61, 1.70)	1.85 (1.69, 2.02)	1.41 (1.34, 1.48)	1.34 (1.29, 1.39)	2.31 (2.28, 2.35)	2.22 (2.11, 2.32)	1.73 (1.68, 1.80)	1.72 (1.63, 1.81)	2.17 (2.15, 2.20)	2.13 (2.04, 2.22)	1.61 (1.57, 1.66)	1.45 (1.41, 1.49)
	6-11	1.92 (1.85, 1.99)	1.95 (1.74, 2.19)	1.89 (1.78, 2.0)	1.67 (1.60, 1.74)	2.59 (2.56, 2.63)	2.37 (2.25, 2.49)	2.16 (2.09, 2.25)	1.67 (1.58, 1.76)	2.50 (2.47, 2.54)	2.30 (2.19, 2.41)	2.08 (2.02, 2.15)	1.77 (1.71, 1.83)
	12-23	1.53 (1.47, 1.60)	1.41 (1.27, 1.57)	1.75 (1.66, 1.84)	1.74 (1.67, 1.81)	1.94 (1.91, 1.97)	1.79 (1.70, 1.88)	1.94 (1.87, 2.01)	1.48 (1.40, 1.57)	1.88 (1.86, 1.91)	1.71 (1.63, 1.79)	1.87 (1.82, 1.93)	1.76 (1.70, 1.82)
	24-59	1.36 (1.29, 1.42)	1.76 (1.54, 2.00)	1.62 (1.55, 1.70)	1.85 (1.78, 1.91)	2.63 (2.56, 2.69)	2.15 (1.95, 2.36)	2.15 (2.03, 2.28)	1.30 (1.18, 1.43)	2.30 (2.25, 2.35)	2.00 (1.86, 2.16)	1.83 (1.77, 1.90)	1.88 (1.82, 1.95)
Obs Unit	0-5	1.73 (1.66, 1.80)	1.29 (1.14, 1.46)	1.24 (1.15, 1.32)	1.16 (1.09, 1.23)	2.02 (1.95, 2.09)	1.83 (1.65, 2.03)	1.57 (1.44, 1.71)	1.72 (1.52, 1.95)	1.88 (1.83, 1.93)	1.58 (1.46, 1.71)	1.36 (1.29, 1.43)	1.21 (1.15, 1.28)
	6-11	1.68 (1.57, 1.79)	1.20 (.98, 1.46)	1.32 (1.19, 1.47)	1.16 (1.07, 1.27)	2.01 (1.93, 2.10)	1.86 (1.65, 2.10)	1.71 (1.55, 1.89)	1.67 (1.47, 1.89)	1.92 (1.85, 1.99)	1.65 (1.49, 1.83)	1.52 (1.42, 1.63)	1.30 (1.21, 1.40)
	12-23	1.42 (1.33, 1.52)	1.04 (.85, 1.26)	1.38 (1.25, 1.52)	1.18 (1.09, 1.28)	1.38 (1.33, 1.43)	1.40 (1.27, 1.56)	1.41 (1.31, 1.52)	1.48 (1.34, 1.65)	1.39 (1.35, 1.43)	1.32 (1.20, 1.44)	1.40 (1.32, 1.49)	1.23 (1.16, 1.32)
	24-59	1.20 (1.11, 1.30)	1.01 (.80, 1.26)	1.21 (1.09, 1.33)	1.07 (1.0, 1.16)	1.14 (1.06, 1.22)	1.18 (.96, 1.45)	1.19 (1.04, 1.35)	1.30 (1.08, 1.55)	1.17 (1.11, 1.23)	1.10 (.95, 1.28)	1.20 (1.11, 1.30)	1.09 (1.02, 1.17)

a. Overall hospital admissions (ICU and inpatient)

© 2024 Suss RJ et al. *JAMA Network Open.*

eTable 4. RSV and Bronchiolitis Hospital, ICU, Inpatient, Emergency Department, and Observation Unit Admissions in Infants and Children <60 Months of Age, Proportion Medicaid, 2015-2022

4A. RSV and Bronchiolitis Hospital Admissions in Infants and Children <60 Months of Age, Proportion Medicaid, 2015-2022[a]

	RSV				Bronchiolitis				RSV+ Bronchiolitis			
	2015-2019	2020	2021	2022	2015-2019	2020	2021	2022	2015-2019	2020	2021	2022
Age (Months)												
0-5	0.61	0.58	0.55	0.52	0.65	0.65	0.61	0.61	0.63	0.61	0.57	0.54
6-11	0.61	0.58	0.57	0.52	0.66	0.64	0.63	0.58	0.64	0.62	0.60	0.54
12-23	0.58	0.56	0.56	0.51	0.58	0.57	0.57	0.57	0.58	0.57	0.57	0.53
24-59	0.56	0.54	0.55	0.51	0.58	0.60	0.55	0.56	0.57	0.57	0.55	0.52

a. Overall hospital admissions (ICU and inpatient)

4B. RSV and Bronchiolitis ICU Admissions in Infants and Children <60 Months of Age, Proportion Medicaid, 2015-2022

	RSV				Bronchiolitis				RSV+ Bronchiolitis			
	2015-2019	2020	2021	2022	2015-2019	2020	2021	2022	2015-2019	2020	2021	2022
Age (Months)												
0-5	0.62	0.58	0.57	0.54	0.68	0.66	0.66	0.64	0.64	0.61	0.59	0.56
6-11	0.61	0.55	0.59	0.54	0.67	0.67	0.67	0.62	0.64	0.62	0.64	0.56
12-23	0.57	0.56	0.58	0.52	0.60	0.57	0.59	0.60	0.59	0.57	0.59	0.55
24-59	0.57	0.54	0.57	0.51	0.60	0.62	0.58	0.61	0.58	0.58	0.58	0.53

4C. RSV and Bronchiolitis Inpatient Admissions in Infants and Children <60 Months of Age, Proportion Medicaid, 2015-2022

	RSV				Bronchiolitis				RSV+ Bronchiolitis			
	2015-2019	2020	2021	2022	2015-2019	2020	2021	2022	2015-2019	2020	2021	2022
Age (Months)												
0-5	0.60	0.58	0.54	0.51	0.64	0.64	0.59	0.59	0.62	0.60	0.55	0.52
6-11	0.61	0.60	0.56	0.52	0.65	0.62	0.61	0.56	0.63	0.61	0.58	0.53
12-23	0.58	0.55	0.55	0.51	0.58	0.57	0.55	0.55	0.58	0.57	0.55	0.53
24-59	0.55	0.54	0.54	0.51	0.57	0.59	0.53	0.52	0.56	0.56	0.54	0.51

© 2024 Suss RJ et al. *JAMA Network Open.*

4D. RSV and Bronchiolitis Emergency Department Admissions in Infants and Children <60 Months of Age, Proportion Medicaid, 2015-2022

	RSV				Bronchiolitis				RSV+ Bronchiolitis			
	**2015-2019**	**2020**	**2021**	**2022**	**2015-2019**	**2020**	**2021**	**2022**	**2015-2019**	**2020**	**2021**	**2022**
Age (Months)												
0-5	0.62	0.65	0.58	0.57	0.70	0.69	0.63	0.63	0.68	0.68	0.62	0.59
6-11	0.66	0.66	0.65	0.63	0.72	0.70	0.68	0.66	0.71	0.70	0.68	0.64
12-23	0.61	0.59	0.64	0.63	0.66	0.64	0.66	0.64	0.65	0.63	0.65	0.64
24-59	0.58	0.64	0.62	0.65	0.72	0.68	0.68	0.68	0.70	0.67	0.65	0.65

4E. RSV and Bronchiolitis Observation Unit Admissions in Infants and Children <60 Months of Age, Proportion Medicaid, 2015-2022

	RSV				Bronchiolitis				RSV+ Bronchiolitis			
	**2015-2019**	**2020**	**2021**	**2022**	**2015-2019**	**2020**	**2021**	**2022**	**2015-2019**	**2020**	**2021**	**2022**
Age (Months)												
0-5	0.63	0.56	0.55	0.54	0.67	0.65	0.61	0.60	0.65	0.61	0.58	0.55
6-11	0.63	0.54	0.57	0.54	0.67	0.65	0.63	0.62	0.66	0.62	0.60	0.57
12-23	0.59	0.51	0.58	0.54	0.58	0.58	0.59	0.57	0.58	0.57	0.58	0.55
24-59	0.55	0.50	0.55	0.52	0.53	0.54	0.54	0.55	0.54	0.52	0.55	0.52

© 2024 Suss RJ et al. *JAMA Network Open.*
