# Supplementary material for: Respiratory Syncytial Virus Hospital-Based Burden of Disease in Children Younger Than 5 Years, 2015-2022
Source: JAMA Netw Open. 2024 Apr 18;7(4):e247125. doi: 10.1001/jamanetworkopen.2024.7125 (PMC12068875; doi:10.1001/jamanetworkopen.2024.7125)
Supplement: Supplement 2. — Data Sharing Statement [file jamanetwopen-e247125-s002.pdf]

Data Sharing Statement

Suss. Respiratory Syncytial Virus Hospital-Based Burden of Disease in Children Younger Than 5 Years, 2015-2022. *JAMA Netw Open*. Published April 18, 2024.
doi:10.1001/jamanetworkopen.2024.7125

Data
Data available: No

Additional Information
Explanation for why data not available: Database access requires training and completion of an agreement with a third party (the Children's Hospital Association) on a per person basis. Typically, access is only granted to individuals affiliated with member hospitals. It is not within the authors' discretion to share this data.
